# Use of Autologous Serum Eye Drops with Contact Lenses in the Treatment of Chemical Burn-Induced Bilateral Corneal Persistent Epithelial Defects

**DOI:** 10.1155/2022/6600788

**Published:** 2022-02-21

**Authors:** Yan-Ming Chen, Wei-Yu Wang, Yen-Chun Lin, Shih-Hao Tsai, Yun-Ting Lou

**Affiliations:** ^1^Department of Ophthalmology, E-Da Hospital, I-Shou University, Kaohsiung, Taiwan; ^2^Department of Ophthalmology, National Taiwan University Hospital, Taipei, Taiwan; ^3^School of Medicine, I-Shou University, Kaohsiung, Taiwan

## Abstract

**Objective:**

We aim to evaluate the clinical effect of combined topical 20% autologous serum eye drops (ASEs) along with silicone–hydrogel soft contact lenses (SCLs) in the treatment of chemical burn-induced bilateral corneal persistent epithelial defects (PEDs) and to review the literature of related studies.

**Methods:**

From January 1, 2017, to December 31, 2019, we conducted a retrospective chart review of 8 patients with chemical burn-induced bilateral corneal PEDs who were unsuccessfully treated with conventional medical therapy and were then treated with combined topical 20% (v/v) ASEs and silicone–hydrogel CLs. The clinical effects and effectiveness of the combined treatment were evaluated.

**Results:**

The bilateral corneal PEDs healed in all sixteen eyes of the eight patients within 2 weeks. The patients did not report any discomfort associated with the combined treatment. Improved ocular comfort/visual acuity and decreased conjunctival injection correlated with healing. No recurrent corneal epithelial breakdown was noted during the 3-month posttreatment follow-up.

**Conclusions:**

The combined treatment of silicone–hydrogel CLs and ASEs can help to stabilize the ocular surface and successfully treat chemical burn-induced bilateral corneal PEDs. It may be considered as an alternative treatment method for patients with bilateral chemical burn-induced corneal PEDs with potential corneal melting.

## 1. Introduction

Chemical (alkali and acid) injury of the conjunctiva and cornea is considered as a true ocular emergency that requires appropriate intervention [1]. Chemical burn to the eye can produce extensive damage to the whole ocular surface and also the corneal subbasal nerve fibers that sometimes lead to poor reepithelialization and closure of corneal epithelium even after emergent and initial management. Chemical burn-induced corneal persistent epithelial defect (PED) is defined as a the failure of rapid corneal reepithelialization and closure within 10-14 days after chemical burn injury, despite conventional treatments such as artificial tear lubricating gel/ointment application or patching [1]. It can sometimes lead to visual impairment and disfigurement in severe cases [1, 2]. Nowadays, treating chemical burn-induced bilateral corneal PEDs is still difficult and challenging, and the surgical treatment method is required occasionally for the management of very severe and recalcitrant cases that are unresponsive to the traditional medical therapy [2]. Many different methods have been used to treat corneal PED; for example, amniotic membrane transplantation (AMT) and temporary or permanent tarsorrhaphy are occasionally indicated to manage recalcitrant patients that are unresponsive to medical therapy. Nowadays, despite various medical and surgical therapies having been proposed and used to treat corneal PEDs, it remains yet difficult and challenging to treat, and the lack of positive response is still commonly observed in the clinical practice [2–5]. In recent years, the strategy for corneal PED treatment by the combination of bandage contact lenses (BCLs) and autologous serum eye drops (ASEs) has been reported to yield satisfactory results in some studies [6–9]. However, combined treatment using BCLs and ASEs for chemical burn-induced bilateral corneal PEDs has been seldom reported. Herein, we describe the therapeutic outcomes in eight patients with chemical burn-induced bilateral corneal PEDs who were successfully treated by a combination of topical 20% ASEs and silicone–hydrogel CLs and also review the literature of related studies.

## 2. Materials and methods

### 2.1. Patients

A total sixteen eyes in 8 patients with corneal PEDs secondary to chemical burn who were treated with a combination of ASEs and silicone–hydrogel CLs at the corneal and external eye disease clinic of the department of ophthalmology of E-Da Hospital, I-Shou University, Kaohsiung city, Taiwan, from January 1, 2017, to December 31, 2019 were retrospectively reviewed. The patients were informed as the advantages, disadvantages, and potential risks and complications of the treatment. The related study content was approved by the Institutional Review Board for Human Studies of the E-Da Hospital, I-Shou University (EMRP-110-004), and the study adhered to the tenets of the Declaration of Helsinki, and written informed consent was obtained prior to the combined therapy. Before attempting combined therapy, several sole agents were administered, including lubricating gel/ointment, artificial tears, preservative-free artificial tears, ASE treatment alone, and CL application alone. Nevertheless, the lesions failed to epithelialize, and the epithelial defects persisted for more than 2 weeks with progressive corneal stromal melting noted.

### 2.2. Combined Autologous Serum and Contact Lens Treatment

During the treatment period, the silicone–hydrogel CLs (PureVision; Bausch & Lomb, Rochester, NY, USA) were applied in all 8 patients along with 20% (v/v) ASEs bihourly during the waking hours. Prophylactic 0.5% levofloxacin ophthalmic eye drops (Cravit; Santen, Osaka, Japan) four times daily were also prescribed topically for prevention of corneal infection secondary to PEDs. The previous medications including artificial tears and preservative-free artificial tears were discontinued. The autologous serum was prepared as described before [8–10]. The progress of corneal epithelial healing was checked and recorded every 1 to 3 days under slit-lamp examination in our clinic until total corneal reepithelialization was visualized. If complete corneal reepithelialization was observed in the clinical visit, the contact lens was then removed immediately, and the patient continued the use of ASEs four times daily in both eyes for 2 more weeks. Patient profiles are detailed in Table 1.

## 3. Results

All sixteen eyes attained complete corneal reepithelialization within 2 weeks of combined silicone–hydrogel CLs and ASEs therapy. Mean time between chemical injuries and start of combined therapy was 81.1 ± 14.9 days (range 56–115). The therapeutic outcomes are summarized in Table 1. During the 3-month follow-up, no side effects were observed in any of the 8 patients and recurrence of the epithelial breakdown was not observed in any of the 16 eyes. Two representative cases of the 8 patients are discussed below.

### 3.1. Case 1 (Patient No. 1)

**Figure 1:**
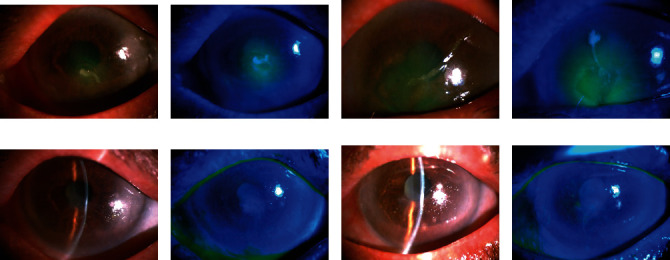
Case presentation. (a and b) Slit-lamp microscopic images of a 78-year-old woman with bilateral nonhealing corneal PEDs measuring 2.0 × 2.0 mm on the right eye under fluorescein staining. (c and d) Corneal PEDs measuring 4.0 × 4.0 mm on the left eye under fluorescein staining. Mild corneal edema associated with the defects and initial corneal melting at the central lesions is also noted on both eyes. (e and f) Five days after initiating combined silicone–hydrogel CL and 20% ASE treatment, the lesion shows reepithelialization, and no corneal epithelial defect is noted on her right eye. (g and h) The lesion is completely reepithelialized on the left eye after twelve days of combined treatment. After total reepithelialization, the CLs are removed and the ASEs are tapered to four times daily for an additional 2 weeks. No recurrence of the epithelial breakdown is noted during a 3-month posttreatment follow-up.

A 78-year-old woman was referred by her general ophthalmologist because of decreased visual acuity, severe pain, and redness in both her eyes for more than 5 weeks. According to her ophthalmologist, she had corneal epithelial defects resulting from accidental exposure to salicylic acid that was mistakenly used instead of eye drops. She denied any prior ocular surgery history but had diabetes mellitus for 12 years. Topical antibiotic eye drops/ointments, preservative-free artificial tears, pressure patching, ASE treatment alone, and CL application alone were attempted, but no improvement was noted by her general ophthalmologist. At our clinic, the initial best-corrected visual acuity (BCVA) of her both eyes was counting fingers at 1 m distance. Slit-lamp examinations revealed bilateral corneal epithelial defects, measuring 2.0 × 2.0 mm on the right eye (Figures 1(a) and 1(b)) and 4.0 × 4.0 mm on the left one (Figures 1(c) and 1(d)). Although the lesions showed no active infectious signs, repeated corneal cultures were performed; however, the culture results were negative after several days of testing. Mild corneal edema associated with the defects and initial corneal melting at the central lesions was also noted on her both eyes. AMT and temporary tarsorrhaphy were recommended initially, but the patient refused any surgery. Therefore, we opted to treat her using topical 20% ASEs bihourly during the waking hours, combined with silicone–hydrogel CL application and prophylactic 0.5% levofloxacin antibiotic eye drops (Cravit; Santen, Osaka, Japan) four times daily on both her eyes. Two days after initiating the combined treatment, the lesions showed signs of reepithelialization. The defects gradually healed and reached complete reepithelialization on day 5 on the right eye (Figures 1(e) and 1(f)) and on day 12 on the left one (Figures 1(g) and 1(h)). The silicone–hydrogel CLs were removed immediately after total reepithelization, but topical ASEs were still used four times daily for 2 additional weeks on her both eyes. Faint central corneal scars developed on her both eyes at the clinical visit 1 month later. The BCVA improved to 20/100 in the right eye and 20/200 in the left one. The patient did not report any discomfort associated with the combined treatment, and no epithelial breakdown was noted again during the 3-month posttreatment follow-up.

### 3.2. Case 2 (Patient No. 3)

**Figure 2:**
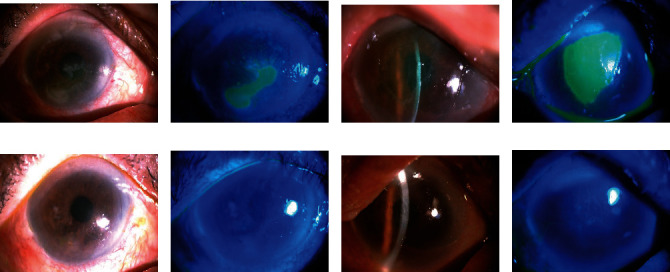
Case presentation. (a and b) Slit-lamp microscopic images of a 69-year-old man with bilateral nonhealing corneal PEDs measuring 4.0 × 3.5 mm on the right eye under fluorescein staining. (c and d) Corneal PEDs measuring 4.5 × 6.0 mm on the left eye under fluorescein staining. (e and f) Seven days after initiating combined silicone–hydrogel CL and 20% ASE treatment, total reepithelialization is achieved on his right eye. (g and h) The lesion heals after 14 days of combined treatment on the left eye. After total reepithelialization, the CLs are removed and the ASEs are tapered to four times daily for an additional 2 weeks. No recurrence of the epithelial breakdown is noted during a 3-month posttreatment follow-up.

A 69-year-old man was referred with a 3-week history of bilateral eye pain, redness, and decreased visual acuity after accidental chemical burns caused by unknown drugs in his both eyes. He denied any prior ocular surgery history and had no underlying systemic disease history. Topical antibiotic eye drops/ointments, preservative-free artificial tears, pressure patching, CL application alone, and ASE treatment alone were attempted, but no improvement was noted by his general ophthalmologist. At our clinic, the initial BCVA of his both eyes was counting fingers at 1 m distance. Slit-lamp examinations revealed bilateral corneal epithelial defects, measuring 4.0 × 3.5 mm on the right eye (Figures 2(a) and 2(b)) and 4.5 × 6.0 mm on the left one (Figures 2(c) and 2(d)). Repeated corneal cultures were performed; however, the culture results were negative after several days of testing. The patient refused any surgical intervention; thus, we tried to treat him by using topical 20% ASEs bihourly during the waking hours, combined with silicone–hydrogel CL application and prophylactic 0.5% levofloxacin antibiotic eye drops (Cravit; Santen, Osaka, Japan) four times daily on his both eyes. Three days after initiating the combined treatment, the lesions showed signs of reepithelialization. The defects gradually healed and reached complete reepithelialization on day 7 on the right eye (Figures 2(e) and 2(f)) and on day 14 on the left one (Figures 2(g) and 2(h)). The silicone–hydrogel CLs were removed immediately after total reepithelization, but topical ASEs were applied four times daily for 2 additional weeks on his both eyes. The BCVA improved to 20/50 in both his eyes at the clinical visit 1 month later. The patient did not report any discomfort associated with the combined treatment during the treatment period, and no recurrent epithelial defect was noted during the 3-month posttreatment follow-up.

## 4. Discussion

According to the previous related studies, PED has many causes such as mechanical injury, chemical burn, toxic agents exposure, inflammation or infections of the ocular surface, or various underlying diseases, including severe dry eye syndrome, neurotrophic keratitis (NK), deficiency of corneal limbal stem cell, and diabetic keratopathy; moreover, it can be very difficult to treat [2, 4, 5]. Corneal PED after ocular chemical burn has been due to severe damage to the corneal limbal stem cells/corneal subbasal nerve fibers and also the release of lytic enzymes, free radicals, inflammatory factors, and other proteases from the polymorphonuclear leukocytes and the healing tissue [1]. It can lead to a vicious cycle of further corneal stromal melting and even corneal perforation without appropriate management. To date, several different methods have been employed to treat corneal PED, but it remains difficult and challenging to treat [2–9]. The first applications of ASEs to support corneal disease treatment were performed in 1975 in corneal alkali injury cases [8]. In recent years, autologous serum, autologous platelet-rich plasma (PRP), and some new eye drop products like and recombinant human NGF (rhNGF) eye drops have been clinically used as an effective topical medication for treating various kinds of ocular surface disorders including PEDs [4, 5, 10, 11]. Autologous serum and natural tear have a very similar composition and nutritional supplements. Autologous serum can provide the human eye surface with many basic nutrient factors for rapid ocular surface reepithelialization. These factors include fibronectin, prealbumin, and neural/epithelial healers such as insulin-like growth factor 1, nerve growth factor, and substance P which may be beneficial in corneal epithelial renewal [10]. Corneal PED is considered as the second stage of NK [12]. The role of nerve growth factor is crucial in NK since it is fundamental in normal ocular surface homeostasis and wound healing [12]. In vivo studies had proved that the NGF concentration increases after ocular injury, and that administration of NGF can accelerate corneal wound healing [13]. Since ASEs harbor neurotrophic factors, ASEs treatment may provide neural healers to the compromised ocular surface in patients with chemical burn-induced corneal PEDs [14]. Like ASEs, BCLs have been considered for delayed epithelial healing and reported as an effective PED treatment method [15]. First, they are thought to decrease corneal epithelial necrosis and desquamation by preventing mechanical stress associated with blinking, thereby accelerating corneal reepithelialization and closure. Second, BCLs could help to form a stable tear film and thus promote corneal repair. Third, BCLs could help to prolong the duration of eye drops, resulting in a stronger therapeutic effect of the eye drops in the combined treatment [6]. The combined use of ASEs and BCLs in the treatment of PED has been reported in several studies [5–8]. These studies are summarized and compared in Table 2. In our previous studies, we also showed that the combination of topical ASEs and BCLs can treat corneal PEDs of varying causes successfully [7, 8]. In our opinion, the ASEs may help accelerate ocular surface healing in patients with corneal PED, and the use of silicone–hydrogel CLs could stabilize the ocular surface and resolve the problem of persistent foreign-body sensation and pain brought about by the corneal PED. In cases of bilateral chemical burn-induced PEDs, the treatment is more challenging because patients with bilateral PEDs usually cannot open both of their eyes due to the persistent foreign-body sensation and severe eye pain. The addition of BCLs could reduce the foreign-body sensation and pain, thus help to resolve this problem [8, 9, 15]. Traditional treatment methods such as bilateral pressure patching, AMT, or tarsorrhaphy would be very inconvenient for the patients because both the eyes are involved. This combined treatment is particularly desirable in that context since it does not involve interventions which would further compromise visual acuity such as AMT or tarsorrhaphy. Therefore, a combination of BCLs and ASEs seems to be a better alternative treatment choice for patients with bilateral PEDs. In our present study, we removed the silicone–hydrogel CLs immediately after total reepithelization but continued to use topical ASEs for an additional 2 weeks in both the eyes, because our previous preliminary study on the therapeutic outcomes of combined topical ASEs with silicone–hydrogel soft CLs in corneal PED showed that the prolonged usage of ASEs four times daily for an additional 2 weeks even after corneal total reepithelialization seemed to effectively decrease the recurrent epithelial breakdown rates [8, 9]. In these eight patients of the study, no recurrence of epithelial defect was noted during the 3-month posttreatment follow-up, which was in accordance with the findings of our previous study [8, 9].

This study appears to be unique in showing that the combined use of silicone–hydrogel CLs and 20% autologous serum eye drops may effectively treat chemical burn-induced bilateral corneal PEDs. However, our study has several limitations. First, in our study, only chemical burn-induced bilateral corneal PEDs were evaluated. Other pathogenesis of bilateral corneal lesions may be included in the future studies to evaluate the effect of the combined treatment for bilateral corneal lesions. Second, we only used 20% (v/v) ASEs in our patients, rather than 50% (v/v), 100% (v/v) ASEs, or other new products, such as autologous platelet-rich plasma (PRP) or recombinant human NGF (rhNGF) eye drops, for the combination treatment. As we know, different concentrations of ASEs or different formula may lead to different clinical effects and effectiveness in these patients. Third, we only retrospectively evaluated the therapeutic effects and effectiveness of the combined treatment in our patients in the present study, further prospective clinical study in the future with comparative groups is needed to confirm our results.

## 5. Conclusions

In summary, our present study showed that the noninvasive combined use of silicone–hydrogel CLs and 20% ASEs is potentially efficacious, well tolerated, and also associated with an improvement of final BCVA, which may be considered as an alternative for the treatment of chemical burn-induced bilateral corneal PEDs.

## Figures and Tables

**Table 1:** Demographic data of the 8 patients of chemical burn-induced bilateral corneal PEDs receiving combined treatments of silicone–hydrogel CLs and 20% autologous serum eye drops.

Case no.	Age (years)	Gender	Mechanisms of chemical injuries	Chemical burn grading^∗^ OD OS	Previous treatment(s) for bilateral PEDs	Lesion size (mm) OD OS	Time between chemical injuries and start of combined treatments (days)	Resolution time^∗∗^ (days) OD OS	BCVA before combined treatments OD OS	Final BCVA OD OS
1	78	F	Acid burns (salicylic acid)	Grade II Grade III	Topical antibiotic eye drops/ointments; preservative-free artificial tears; pressure patching; ASE treatment alone; CL application alone	2.0 × 2.0 4.0 × 4.0	87	5 12	CF/1 m CF/1 m	20/100 20/200
2	59	M	Acid burns (salicylic acid)	Grade II Grade III	Topical antibiotic eye drops/ointments; artificial tears; lubricating gel/ointment; pressure patching; ASE treatment alone; CL application alone	2.0 × 2.0 4.5 × 5.0	67	5 12	20/400 CF/1 m	20/40 20/50
3	69	M	Unknown drugs	Grade II Grade III	Topical antibiotic eye drops/ointments; preservative-free artificial tears; pressure patching; CL application alone; ASE treatment alone	4.0 × 3.5 4.5 × 6.0	63	7 14	CF/1 m CF/1 m	20/50 20/50
4	63	F	Alkali burns **(**ammonia)	Grade III Grade III	Topical antibiotic eye drops/ointments; artificial tears; lubricating gel/ointment; CL application alone; ASE treatment alone	3.5 × 3.5 3.5 × 4.0	98	7 10	CF/1 m CF/1 m	20/100 20/100
5	57	M	Acid burns (sulfuric acid)	Grade III Grade III	Topical antibiotic eye drops/ointments; preservative-free artificial tears; pressure patching; CL application alone; ASE treatment alone	4.0 × 4.5 4.5 × 5.0	84	7 7	CF/1 m CF/1 m	20/50 20/50
6	52	F	Acid burns (hydrofluoric acid)	Grade II Grade III	Topical antibiotic eye drops/ointments; preservative-free artificial tears; CL application alone; ASE treatment alone	2.0 × 2.0 3.0 × 4.0	79	5 10	20/400 CF/1 m	20/50 20/100
7	60	F	Alkali burns **(**ammonia)	Grade III Grade III	Topical antibiotic eye drops/ointments; preservative-free artificial tears, pressure patching; CL application alone; ASE treatment alone	4.5 × 4.5 4.5 × 5.0	115	7 9	CF/1 m CF/1 m	20/100 20/100
8	55	M	Unknown drugs	Grade II Grade II	Topical antibiotic eye drops/ointments; preservative-free artificial tears; ASE treatment alone; CL application alone	3.5 × 3.5 3.5 × 4.0	56	5 6	CF/1 m CF/1 m	20/50 20/50

^∗^Roper-Hall classification for the severity of ocular surface burns. ^∗∗^Time since starting combined therapy. Abbreviation: M: male; F: female; OD: right eye; OS: left eye; BCVA: best-corrected visual acuity; CLs: contact lenses; ASE: autologous serum eye drop; CF: counting fingers.

**Table 2:** Studies on the combination of bandage contact lenses and autologous serum eye drops for corneal PED treatment.

	This study	Schrader et al. [6]	Choi and Chung [7]	Lee et al. [8]	Wang et al. [9]
Study design	Retrospective study	Retrospective study	Prospective study	Prospective study	Retrospective study
Patient number	8	5	8	21	12
Lesion eye	Bilateral	Four unilateral One patient treated two times in 2 years	Unilateral	Unilateral	Unilateral
Pathogenesis of PED	Chemical burn	Rheumatoid sterile corneal ulcer, neurotrophic keratopathy, and partial limbal stem cell deficiency	Sjögren-type dry eye syndrome, graft-versus-host disease, toxic keratitis, limbal cell deficiency, superior limbic keratoconjunctivitis, and neurotrophic keratitis	Chemical burn with partial limbal stem cell deficiency, corneal epithelial debridement caused by mechanical debridement during pars plana vitrectomy, postinfectious, and neurotrophic keratopathy	Postinfectious
Type of contact lens	Silicone–hydrogel CLs	FDA group IV hydrogel lenses	Silicone–hydrogel CLs	Silicone–hydrogel CLs	Silicone–hydrogel CLs
Autologous serum concentration	20% (v/v)	20% (v/v)	50% (v/v)	20% (v/v)	20% (v/v)
Frequency of autologous serum eye drop application	Every 2 hours	8 times a day	Every 2 hours	Every 2 hours	Every 2 hours
Treatment response	All healed in 2 weeks	All healed in 14.2 ± 8.9 days	All healed in 11.8 ± 4.9 days	All healed in 3 weeks	All healed in 2 weeks

Abbreviations: PED: persistent epithelial defect; CLs: contact lenses; FDA: US Food and Drug Administration; v/v: volume/volume percent.

## Data Availability

The data that support the findings of this study are available from the corresponding author, Y. C., upon reasonable request.
